# Malaria in Highlands of Ecuador since 1900

**DOI:** 10.3201/eid1804.111267

**Published:** 2012-04

**Authors:** Lauren L. Pinault, Fiona F. Hunter

**Affiliations:** Brock University, St. Catharines, Ontario, Canada

**Keywords:** malaria, parasites, Ecuador, Andes Mountains, Anopheles pseudopunctipennis, Anopheles albimanus, Anopheles punctimacula, mosquitoes, highlands

## Abstract

A recent epidemic of malaria in the highlands of Bolivia and establishment of multiple *Anopheles* species mosquitoes in the highlands of Ecuador highlights the reemergence of malaria in the Andes Mountains in South America. Because malaria was endemic to many highland valleys at the beginning of the 20th century, this review outlines the 20th century history of malaria in the highlands of Ecuador, and focuses on its incidence (e.g., geographic distribution) and elimination from the northern highland valleys of Pichincha and Imbabura and the role of the Guayaquil to Quito railway in creating highland larval habitat and inadvertently promoting transportation of the vector and parasite. Involvement of control organizations in combating malaria in Ecuador is also outlined in a historical context.

## Abstract 2

Eliminated after 1950, malaria may be reemerging in a new region.

Some authors have speculated that *Anopheles* mosquitoes may begin transmitting malaria parasites (*Plasmodium* spp.) at higher altitudes in the South American Andes because of climate change (*1**,**2*). In contrast, highland malaria in Africa has more often been attributed to land use alterations, malaria treatment resistance, changes in vector control measures, and human migration into foothill and mountainous regions (*3*). Before 2004, a short-lived epidemic of *P*. *vivax* malaria was recorded in a village in Bolivia at an altitude of 2,300 m that was transmitted by *Anopheles pseudopunctipennis* Theobald mosquitoes (*4*). Multiple anopheline malaria vectors have also become established in the highlands of Ecuador (*5*).

In this review, we summarize documented cases of highland malaria that occurred in Ecuador during the early 20th century. We define the term highland malaria to mean all malaria that occurs in regions with steep topography. Using geographic information systems (ArcGIS version 10; ESRI, Redlands, CA, USA) and tabulated data from historical sources, we reconstruct the geographic extent of malaria incidence during several periods of interest. We also outline malaria control efforts and attempts at malaria elimination for Ecuador during the 20th century and at the beginning of the 21st century.

## Malaria in Ecuador at the Beginning of the 20th Century

Although malaria was prevalent on the coast of Ecuador at the beginning of the 20th century, it was considered by public health officials to be a minor problem (*6*). Until 1908, Guayaquil on the coast of Ecuador was affected by the constant menace of mosquitoes transmitting yellow fever, and flea-borne bubonic plague reoccurred regularly in all areas of the city and surrounding countryside (*6**–**8*). Because Guayaquil had an image of being an unhealthy major port city, officials in Ecuador signed an international sanitation convention in 1906 to combat outbreaks of yellow fever, bubonic plague, and cholera (*9*). Under terms of the convention, officials were to take measures to prevent ongoing transmission of these diseases, including the use of mosquito screens on windows and doors of hospitals (*9*). During that time, officials also experimented with the use of mosquito larvae–eating fish as a biocontrol method in an attempt to control yellow fever (*8*).

In 1908, the public health movement became active in Ecuador, and a special sanitary commission was formed in Guayaquil (*7**,**8*). At that time, malaria was still considered a minor health problem and many residents allowed mosquitoes to bite them to provide them with long-term immunity to malaria (*10*). Malaria prevention measures included bed nets, window and door screens, and anopheline larval habitat destruction (*8**,**10*). Several medical entomologists became active during this period in Ecuador. These entomologists included the French entomologist Paul Rivet, and the Ecuadorian entomologists F.R. Campos, Luis León, and J. Rodríguez (*11*).

In 1919, many physicians in Ecuador began to receive training in foreign countries, particularly in the United States through grants from the Rockefeller Foundation, in an attempt to eliminate yellow fever and malaria from Ecuador (*6**,**8**,**12*). With the elimination of yellow fever in 1920, attention inevitably turned to malaria, which still accounted for a large percentage of deaths on the coast of Ecuador (*13*). By 1940, malaria still remained a priority and was the second leading cause of death in Ecuador after whooping cough (*7*). At the time, it was recommended that a campaign against malaria should be initiated throughout the entire country (*7*).

## Highland Malaria during Construction of Guayaquil to Quito Railway (1890–1945)

**Figure 1:**
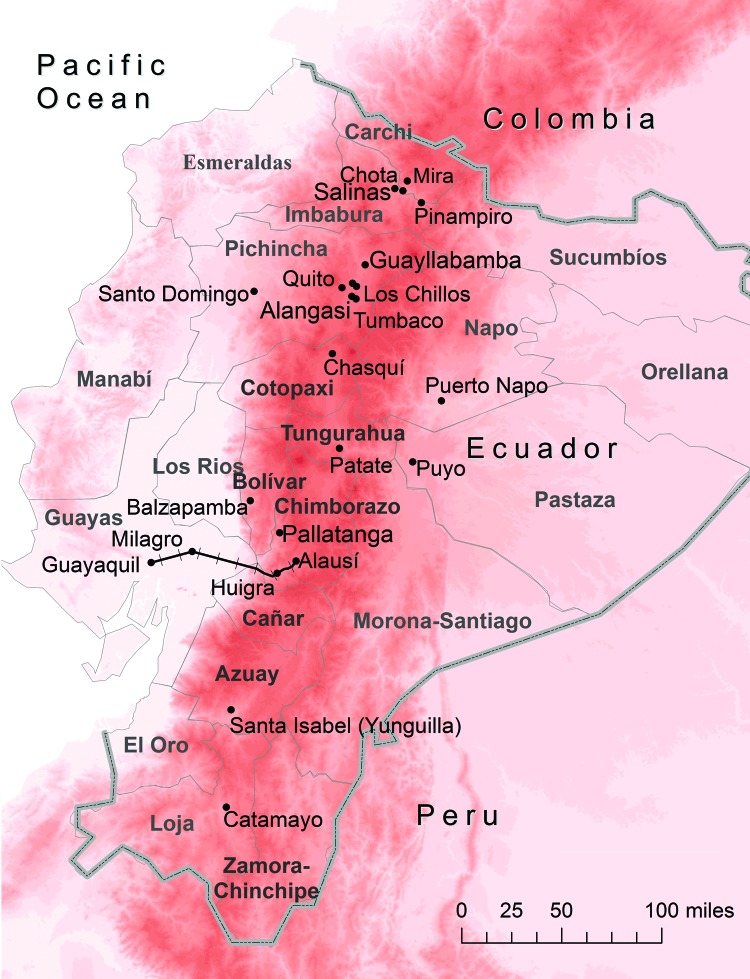
Ecuador showing elevation (red shading), provinces (thin gray lines and **boldface**), country border (thick gray line), and 15 cities/valleys (black dots). Approximate location of the historic railway between Guayaquil and Alausí is indicated by black railroad tracks, and increasing altitude is indicated by darker shades of red. Map was constructed by using ArcGIS version 10 (ESRI, Redlands, CA, USA).

In 1886, construction began on the railway that was to link some of the low-altitude regions of the country near Guayaquil to highland regions and eventually Quito (altitude 2,800 m) (*14*). The railway was constructed on a route that began in Guayaquil (at sea level), passed through Milagro, and followed the valley bottom up toward Huigra, in Chimborazo (altitude 1,250 m). After Huigra, the railway continued higher toward Alausí, Chimborazo (altitude 2,340 m), after climbing the infamous Devil’s Nose switchbacks (*14*) (Figure 1).

When construction of the railway reached an elevation of 200 m, workers reported bites from an unidentified bush-dwelling, flying insect and many subsequently died of high fevers (*15*). At higher altitudes, workers began to report fevers attributed to malaria (*14*). In 1906, the Guayaquil newspaper *Grito del Pueblo* reported that railway workers affected by fevers were removed from the construction site and brought to the highland village of Chasqui, Pichincha, for recovery (*16*). The following quotation from Daniel Barragán, one of the engineers for the railway, provides strong evidence that mosquitoes (Culicidae) were present at worksites: “The mosquitoes were our eternal companions, during all of the night, their melodious and incessant humming many times did not let us find sleep” (translated into English by L.L.P.) (*14*).

Meitzner described treating many of the railway workers for malaria during 1911 (*10*). In the winter of that year, the incidence of malaria was so great among workers that construction halted completely (*10*). Patients were usually brought to higher altitude towns such as Huigra for treatment because there were insufficient medical facilities at lower altitudes (*10*). Patients with malaria among the railway workers were treated by Meitzner by using a combination of castor oil and quinine and a diet that excluded meat (*10*). Before operation of the railway, transportation between the coast and highland regions was limited. Therefore, during the early operation of the railway to Quito, the malaria parasite could have been repeatedly introduced by infected passengers and workers to higher altitude regions, including the valleys around the city of Quito (*10*).

**Figure 2:**
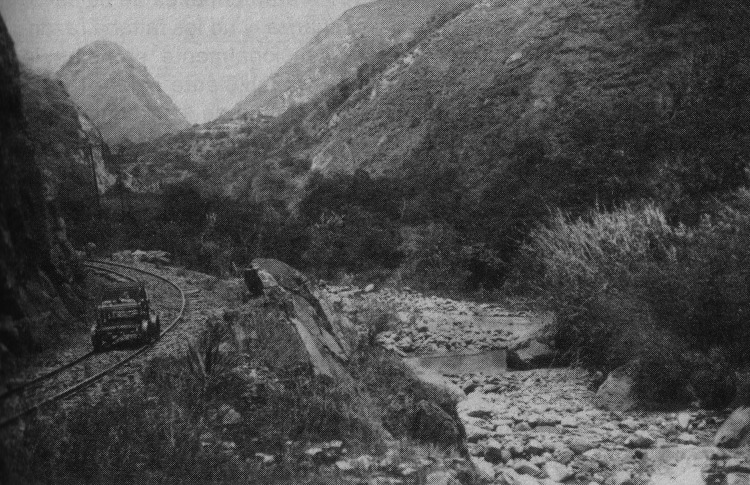
Railway construction at base of the Devil’s Nose switchbacks, Ecuador, showing railway on the left and stone-lined riverbed on the right, where several pools can be seen (likely formed by falling rocks from construction), which would likely have provided suitable habitat for *Anopheles pseudopunctipennis* larvae. Photograph: Historical Archive of Banco Central and García Idrovo (*14*).

The presence of malaria rather than other similar febrile illnesses in railway workers is further supported by multiple collections of *An. pseudopunctipennis* larvae (the highland malaria vector) in the Chiripungo Valley, near Alausí, Chimborazo (altitude <2,400 m) (*17*). As early as 1911, Meitzner made recommendations to railway engineers to construct drainage ditches along the sides of the tracks to prevent establishment of additional larval habitats in the pools that formed there (*10*). Despite the efforts of Meitzner, *An. pseudopunctipennis* mosquitoes remained in highland valleys of Chimborazo along the railway at least into the mid 1940s. In 1943, Levi Castillo collected *An. pseudopunctipennis* larvae along railway tracks up to an altitude of 1,250 m, and in 1944, he collected larvae in pools associated with rivers in the towns of Huigra and Sibambe, Chimborazo (*17*). These entomologic collections are consistent with the epidemiology of continued malaria transmission; in 1944, a total of 154 cases of malaria among 864 residents were documented in Huigra (*18*). Aside from habitats associated with the railway line, as shown in Figure 2, partial blockage of the river below the newly built tracks along Devil’s Nose would probably have produced suitable pools for *An. pseudopunctipennis* larval habitat.

Trains were a likely carrier for continued introduction of anopheline mosquitoes into highland regions from the coast (*17**,**19*). At Milagro station, adult mosquitoes were observed to fill train cars bound for higher elevations (*19*). Levi Castillo found pools of water in ceiling portions of trains, which served as mobile larval habitats for anopheline mosquitoes (*17*). At higher elevations (>1,100 m), *An. pseudopunctipennis* mosquitoes were documented as the sole vector, although *An. albimanus* Wiedemann larvae were collected at lower altitudes along the railway (*18*). Trains likely served to introduce anopheline mosquitoes to highland regions until the 1960s and 1970s, when the railway fell into disuse (*15*).

## Highland Malaria Foci in Ecuador (1900–1950)

Malaria in highland regions of the northern Andes was not exclusive to Ecuador and was found in Colombia in the Cauca, Manizales, Cali, and Medellín Valleys, and in Peru in the Rimac, Urubamba, and Laurin-Orcocota Valleys (*20*). Although malaria became more readily studied and possibly more widespread in Ecuador during the 1940s, the vector mosquito *An. pseudopunctipennis* was likely present for a much longer period in highland regions (*21*). The malaria parasite is believed to have been introduced to a handful of highland valleys in the 1800s because there are no records of it before that time (*22*).

In 1905, students at the University of Guayaquil listed the following highland valleys in Ecuador to which malaria was endemic: Imbabura, Chota and Pinampiro valleys in Pichincha; Tumbaco and Guayllabamba Valleys in Tungurahua; the Patate Valley; and the Yunguilla Valley in Azuay (*23*) (Figure 1). All of these valleys except Patate were regarded as regions to which malaria was endemic into the 1940s (*24*). In almost every highland valley, *Plasmodium vivax* was implicated as the only malaria parasite with *An. pseudopunctipennis* mosquitoes as vectors (*11**,**24*).

Highland malaria was widespread in the early 1940s when it appeared to reach its widest distribution (*24*). In addition to the valleys listed above and highland valleys in Chimborazo associated with the railway, malaria transmission was observed in Imbabura (Mira Valley and Salinas), Pichincha (widespread in all highland valleys), Cañar (all valleys at an elevation <2,500 m), Chimborazo (Pallatanga Valley), Azuay (Yunguilla Valley), and Loja (Catamayo Valley) (*11**,**24*) (Figure 1).

In 1938, Hanson and Montalvan documented a new epidemic of *P. vivax* and *P. falciparum* malaria in Balzapamba, Bolívar (population 700), in an orange-growing region at an elevation of 650 m (*25*) (Figure 1). Residents had reportedly never experienced malaria until 1935, although they lived near (≈10 km away) the malaria-endemic coastal plain. In 1935, an earthquake and associated landslides diverted the course of the main river, and an open canal was constructed to provide the town with drinking water (*25*). Throughout their search, Hanson and Montalvan were able to locate only *An. pseudopunctipennis* larvae in the open canal and in the algae-covered pools, which formed on the edges of the newly-diverted river (*25*). This epidemic highlights the scarcity of available larval habitat in steep topography regions and the probable role of river pools and human-made canals as habitat for anopheline larvae in highland regions.

Although *An. albimanus* mosquitoes have traditionally been considered low-altitude (<300 m) vectors, they were identified as the main malaria vector in an epidemic in the Yunguilla Valley in Azuay (altitude ≈1,500 m) in the late 1940s (*17**,**18*). *An. pseudopunctipennis* mosquitoes were collected from higher-altitude towns in Azuay, such as Santa Isabel, during the 1940s (*18*). DDT was just beginning to be used at that time in Ecuador and was successfully applied in the 1940s to the Yunguilla Valley (*19*). During that time, 5% DDT in a solution of kerosene was applied inside homes and to larval habitats (*26*).

Little research was conducted in southern Ecuador, although there were confirmed malaria cases in the Chota and Pinampiro Valleys in in Imbabura; the Tumbaco and Guayllabamba Valleys in Pichincha; the Patate Valley in Tungurahua; and the Yunguilla Valley in Azuay (*18*). Montalvan reported his unconfirmed belief that the main vector in Catamayo might be *An. punctimacula* Dyar mosquitoes, which were otherwise not implicated in highland malaria transmission during the 1940s in Ecuador (*18*). Similarly, there are few reports of malaria in highland parts of the Amazonian side of the Andes during the early 20th century, likely because the region was sparsely settled. Even in the lower altitude Ecuadorian Amazon communities such as Puyo and Napo (presently Puerto Napo) (altitude 700–900 m), the residents reported no cases of malaria (*27*) (Figure 1).

## Highland Malaria in Northern Valleys (Pichincha and Imbabura) (1940–1950)

**Figure 3:**
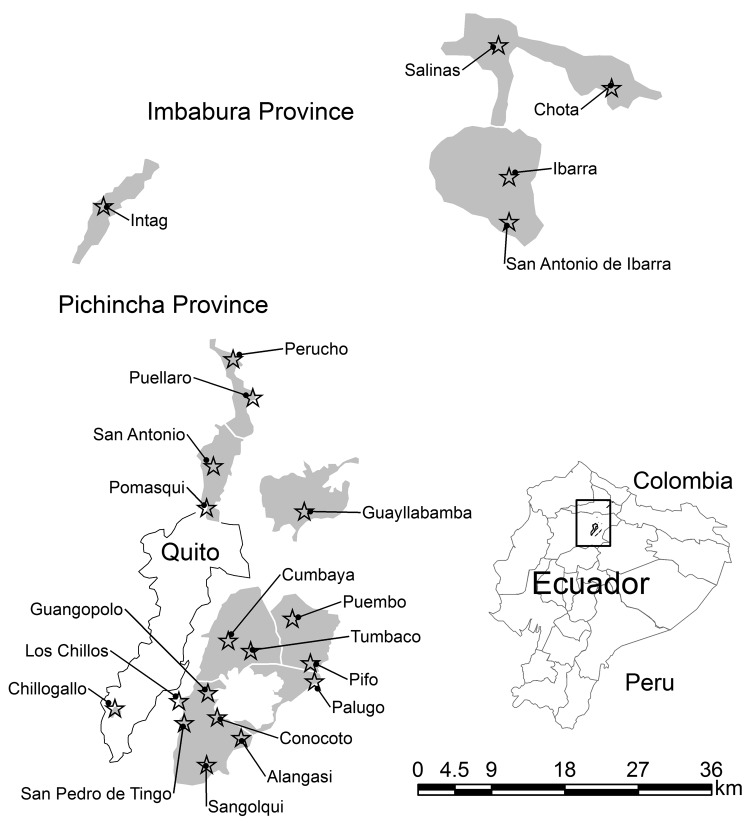
Probable extent of highland valley malaria incidence (shaded areas) during the early 1940s in Ecuador. Stars indicate approximate location of original towns to which malaria was reported as endemic, judged by the presence of the historical town square in Google Earth satellite imagery (Google, 2010). Shading was determined by the valley bottom with an affected town up to an altitude of 2,500 m. Inset: Approximate location of region in Ecuador. Data were obtained from Levi Castillo (*17*), Montalvan (*18*), and Levi Castillo (*21*).

Highland malaria in the northern valleys of Ecuador was well documented during its most widespread period (1940–1950) (*18**,**24*). Malaria was reported from valleys in Imbabura and Pichincha Provinces, although it never reached the city of Quito (altitude 2,800 m) (*24*). On the basis of valleys affected and maximum altitudes recorded for anopheline species, the probable extent of highland malaria in the northern valleys during its peak is shown in Figure 3.

Before the widespread occurrence of malaria in the 1940s, Guayllabamba, Pichincha, was considered an area to which malaria was endemic (*18**,**21**,**22*). Gradually, the vector and parasite spread to other valleys, reaching Tingo and Alangasi by 1917 (*21*) (Figure 1, Figure 3). The spread of malaria may have been in part caused by an exodus of citizens from Guayllabamba during the maximum incidence of the disease (*28*). When malaria became more widespread in the 1940s, the government in Ecuador brought in the US malariologist Henry Hanson, who identified *An. pseudopunctipennis* mosquitoes as the only vectors (*28*). The maximum altitude of the species was estimated to be 2,500 m–2,700 m, although they have since been observed at 3,200 m (*11**,**21**,**26*). *An. pseudopunctipennis* larvae were collected from clean, sunlit, rocky pools associated with rivers, springs of water, irrigation ditches, and hoof-prints from horses (*17**,**18**,**20**,**21*). Entomologists also noted a strong larval association with spirogyra algae (*18**,**21**,**26*).

**Figure 4:**
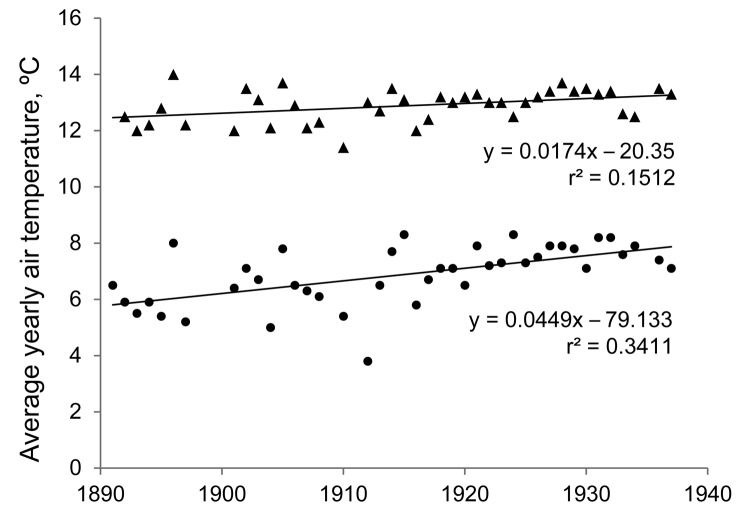
Increase in yearly average daily temperature during a 24-hour period (▲) and average minimum nightly temperature (●) in Quito, Ecuador, 1891–1937, leading up to years of observation of highland malaria in valleys surrounding Quito. Although average temperature only increased at a rate of 0.017°C/year, minimum nightly temperature, which may be more essential for survival of *Anopheles* spp. species, increased at a rate of 0.045°C/year. Data were obtained from the Astronomical and Meteorological Observatory of Quito (*30*).

Although the expansion of *Anopheles* mosquito distribution is often attributed to land use change, highland valleys of northern Ecuador have been cleared and continuously farmed since pre-Colombian times (*29*). The reported use of river edges as habitats also makes land use change unlikely to be the sole explanation (*17**,**18*). Spread of the parasite and vector may also be attributed to meteorologic causes, especially an increase in minimum temperatures, which might otherwise limit parasite or vector development. Increases of 0.5°C in average daily temperature and 1.3°C in minimum nightly temperature were observed in Quito during 1900–1930 (Figure 4). Therefore, meteorologic factors may have caused the increased range of highland malaria before 1940.

A widespread campaign began in 1940 to eliminate malaria from highland valleys of Pichincha and Imbabura (*28*). Malaria was eliminated in the Los Chillos Valley by the *Servicio Antipalúdico del Valle de los Chillos*, led by Jaime Rivadeneira, and assisted by Carlos A. Marín and Benjamín Wandemberg (*11**,**17**,**21**,**24*). A field laboratory was set up in San Pedro de Tingo to lead an initial systematic larval habitat inspection of the entire valley (*21*). All pools of water used by anopheline larvae were drained by the construction of dikes and sprayed with crude oil mixed with kerosene and occasionally DDT (*17**,**21**,**28*). Smaller pools were filled with earth, and residents were provided with chemical repellent for personal use (*17**,**20*). The campaign was deemed a success and malaria did not return in subsequent years (*21*).

## Human Colonization of Coastal Foothill Tropical Forests (1950–1970)

A map of malaria incidence published in 1950 shows the greatest incidence in Ecuador to be in the northern coastal region and *An. albimanus*, *An. pseudopunctipennis*, and *An. punctimacula* mosquitoes to be the most common vectors (*31*). The foothills of the northern coast were sparsely populated and land was not substantially developed before 1950, because the region was covered in dense tropical forest with limited access (*32*). However, roads were built linking Quito to the coast in the late 1940s, and settlers moved into the region, forming Santo Domingo (presently Santo Domingo de los Tsáchiles) (altitude ≈500 m) (*32*). Settlers converted wide swathes of forest to maize, rice, cocoa, and coffee plantations for trade in Quito and in port cities (*32*). Seasonal workers from the highlands migrated into the region to work during summers (*32*). The first major epidemic of malaria was reported in 1958 (*32*). Land conversion likely provided sunlit habitat, which may have been more suitable for some species of *Anopheles* mosquitoes. Also, the immigration of large groups of highlanders lacking immunity likely contributed to an epidemic in 1958 and to subsequent epidemics.

## History of Malaria Elimination/Control Efforts in Ecuador

Although there were several regional public health organizations addressing malaria on the coast of Ecuador, the National Institute of Hygiene and Tropical Medicine Leopoldo Izquieta Pérez was formed in 1940 (*33*). In 1944, Ecuador had the largest available hospital facilities of any country in Latin America. Physicians were trained by the Pan American Sanitary Bureau with funding from the Rockefeller Foundation, and new public health laboratories were constructed (*34**,**35*). In 1948, the *Servicio Nacional Antimalárico* was formed to campaign against malaria, especially on the coast, and to organize DDT spray operations twice a year (*22*). In the early 1950s, US organizations led efforts to eradicate malaria from malaria-endemic countries, although insecticide resistance was beginning to appear and slow the eradication progress (*36*). By 1956, Ecuador was considered to be in an early attack phase of an eradication program (*36*).

After a recommendation from the Pan American Sanitation Committee, the *Sistema Nacional de Eradicación de Malaria* (SNEM) was founded on July 21, 1956 (*13**,**22*). Its focus was to prevent insect-borne diseases through vector control, mainly through use of chemical insecticides and larval habitat elimination, and public education through school visits, interviews, and community meetings (*13*). In addition to malaria, SNEM has monitored and controlled Chagas disease, dengue fever, onchocerciasis, yellow fever (in Amazonia), and leishmaniasis in Ecuador (*13*).

**Figure 5:**
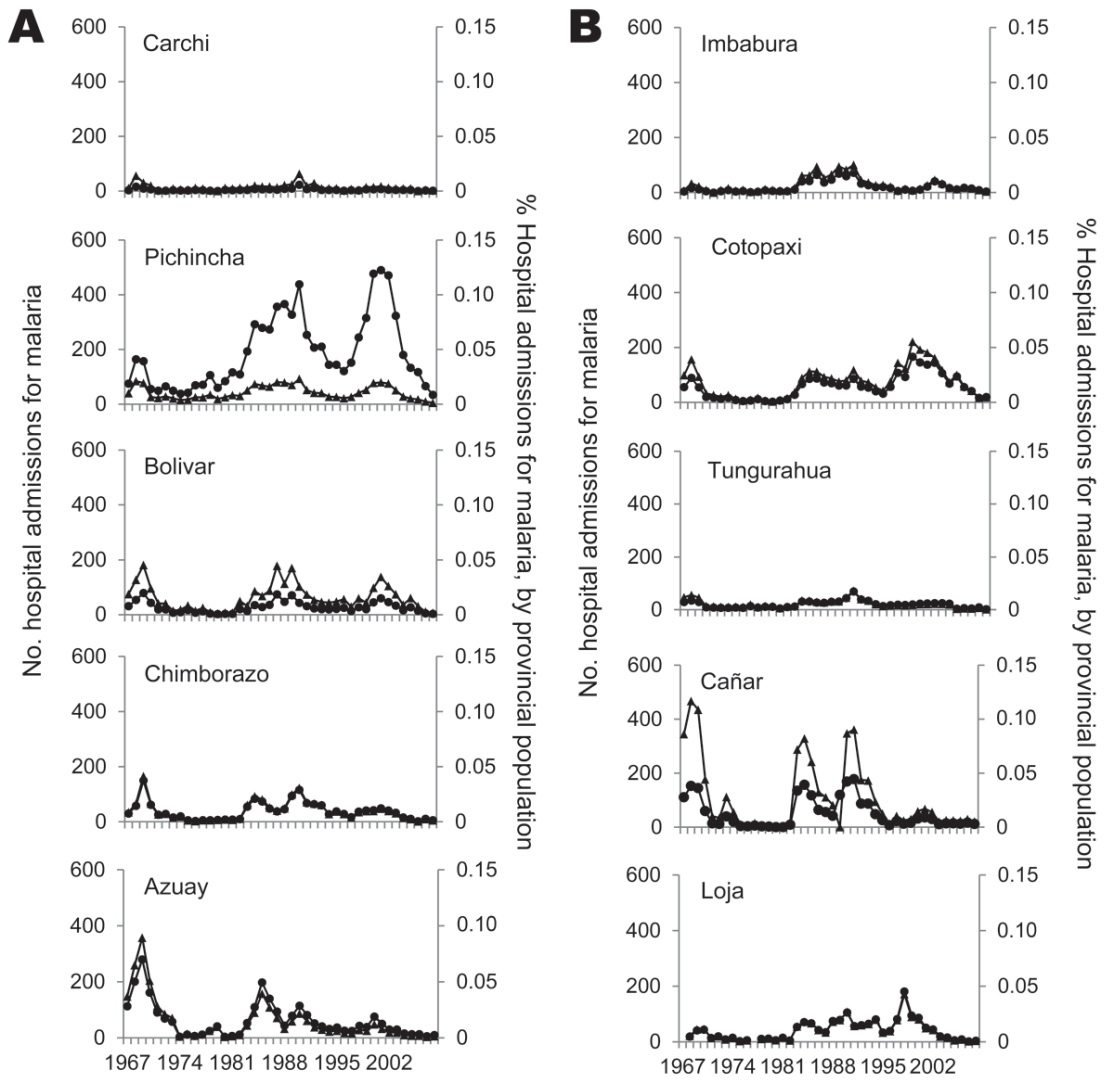
Number of hospital admissions for malaria (▲) per year for each province in the sierra region of Ecuador and percentage of admissions for malaria, by provincial population (●). Data were obtained from the Instituto de Nacional Estatisticas y Censos (*38**,**39*).

**Figure 6:**
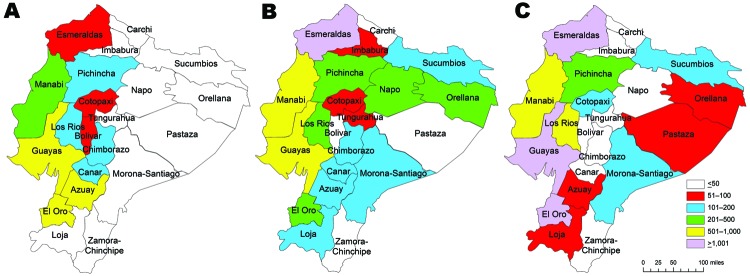
Number of hospital admissions for malaria in each province of Ecuador in peak malaria years of A) 1969, B) 1990, and C) 2000. Data were obtained from the Instituto de Nacional Estatisticas y Censos (*38*).

Success of the SNEM in combating malaria has been closely associated with its variable levels of funding. During 1957–1959, dieldrin was sprayed inside houses on a continuous schedule, but was regularly underdosed and therefore not effective (*37*). During 1961–1965, DDT was applied to houses under the direction of the US Agency for International Development (USAID) and the Pan American Health Organization with greater success (*37*). Funding for medical entomology research was so poor and unreliable that R. Levi Castillo, who had previously documented many cases of highland malaria, renounced his post at the University of Guayaquil and burned his books in protest (*33*). By the late 1960s, USAID funding had decreased substantially, resulting in a subsequent epidemic (*37*). Azuay and Cañar, and to a lesser extent Pichincha and Chimborazo, had a small increase of malaria cases in low-lying valleys (Figure 5). Similarly, in 1969 during another peak year, these highland provinces and most areas of the coast of Ecuador were affected by malaria (Figure 6, panel A).

USAID funding was reinstated in 1973 through a reinvestment with the SNEM (*22**,**37*). However, by 1980, the SNEM was considered operational and no longer relied on international funding (*37*). During an assessment in 1983, the SNEM was deemed to be a capable department but with some financial concerns, such as having an aging fleet of boats and jeeps, and a residual house spray schedule of 3 times a year rather than the recommended 4 times (*37*).

Through the latter half of the 1980s, some malaria cases were reported in highland provinces of Pichincha, Cotopaxi, Bolívar, and to a lesser extent, Chimborazo and Loja (Figure 5). Tungurahua, Carchi, and Imbabura Provinces reported only occasional malaria cases (Figure 5). During 1 of the peak years (1990), there was widespread malaria along the coast, in Amazonia, and in highland provinces (although perhaps only in lower-altitude regions of these provinces) (Figure 5; Figure 6, panel B). Again in 2000, widespread malaria was observed in the coastal and Amazonian areas of Ecuador (Figure 6, panel C), but only Pichincha and to a lesser extent Cotopaxi and Bolívar observed an increase in cases in the highlands (Figure 5, Figure 6). However, despite occasional cases of malaria, the SNEM reported a steady decrease in malaria in Ecuador during the past 20 years, likely as a response to efforts of various programs that have been more recently implemented in the country (*13*).

## Conclusions

Malaria became more widespread in northern highland regions of Ecuador during 1900–1940 but was subsequently eliminated from these regions through habitat elimination and use of chemical insecticides (*21*). In Chimborazo during 1900–1950, malaria spread into highland valleys along the railway linking Guayaquil and Quito (*10**,**17**,**18*). Although there have likely been a few highland epidemics since the 1940s, only 1 report in 1991 documented the presence of *An*. *pseudopunctipennis* mosquitoes in river-associated habitats of Guayllabamba (*28*). To effectively monitor establishment of highland malaria vectors, a focus on historically malaria-endemic highland valleys may be needed. Anopheline habitats in areas with steep topography are expected to differ from those in flat, low-altitude regions. Therefore, these differences will necessitate further study of local dynamics of mosquito ecology, meteorologic variables, and transmission cycles.
